# Identification of hub glycolysis-related genes in acute myocardial infarction and their correlation with immune infiltration using bioinformatics analysis

**DOI:** 10.1186/s12872-024-03989-7

**Published:** 2024-07-10

**Authors:** Xiaoqing Zhang, Lina Zhang, Ya Gao, Zhangyu Liu, Kaizheng Gong

**Affiliations:** 1 Department of Cardiology, Affiliated Hospital of Yangzhou University, No. 368, Hanjiang Middle Road, Yangzhou 225000, Jiangsu China; 2 School of Medicine, Yangzhou University, No. 136, Jiang yang Middle Road, Yangzhou 225000, Jiangsu China; 3 Department of Cardiology, Affiliated Hospital of Nantong University, No.20, Xisi Road, Nantong 226001, Jiangsu China

**Keywords:** Acute myocardial infarction, Glycolysis, Immune infiltration, CIBERSORT, Nomogram, ROC curves

## Abstract

**Purpose:**

Glycolysis and immune metabolism play important roles in acute myocardial infarction (AMI). Therefore, this study aimed to identify and experimentally validate the glycolysis-related hub genes in AMI as diagnostic biomarkers, and further explore the association between hub genes and immune infiltration.

**Methods:**

Differentially expressed genes (DEGs) from AMI peripheral blood mononuclear cells (PBMCs) were analyzed using R software. Glycolysis-related DEGs (GRDEGs) were identified and analyzed using the Database for Annotation, Visualization, and Integrated Discovery (DAVID) for functional enrichment. A protein–protein interaction network was constructed using the STRING database and visualized using Cytoscape software. Immune infiltration analysis between patients with AMI and stable coronary artery disease (SCAD) controls was performed using CIBERSORT, and correlation analysis between GRDEGs and immune cell infiltration was performed. We also plotted nomograms and receiver operating characteristic (ROC) curves to assess the predictive accuracy of GRDEGs for AMI occurrence. Finally, key genes were experimentally validated using reverse transcription-quantitative polymerase chain reaction (RT-qPCR) and western blotting using PBMCs.

**Results:**

A total of 132 GRDEGs and 56 GRDEGs were identified on the first day and 4–6 days after AMI, respectively. Enrichment analysis indicated that these GRDEGs were mainly clustered in the glycolysis/gluconeogenesis and metabolic pathways. Five hub genes (*HK2, PFKL, PKM, G6PD*, and *ALDOA*) were selected using the cytoHubba plugin. The link between immune cells and hub genes indicated that *HK2, PFKL, PKM*, and *ALDOA* were significantly positively correlated with monocytes and neutrophils, whereas *G6PD* was significantly positively correlated with neutrophils. The calibration curve, decision curve analysis, and ROC curves indicated that the five hub GRDEGs exhibited high predictive value for AMI. Furthermore, the five hub GRDEGs were validated by RT-qPCR and western blotting.

**Conclusion:**

We concluded that *HK2, PFKL, PKM, G6PD*, and *ALDOA* are hub GRDEGs in AMI and play important roles in AMI progression. This study provides a novel potential immunotherapeutic method for the treatment of AMI.

**Supplementary Information:**

The online version contains supplementary material available at 10.1186/s12872-024-03989-7.

## Introduction

The most common cause of acute myocardial infarction (AMI) is insufficient coronary blood flow, leading to myocardial necrosis and scar formation, which eventually leads to cardiac insufficiency. In recent years, the popularity of vascular revascularization therapy has greatly improved the survival rate of patients with AMI; however, it also increases the probability of ventricular malremodeling and the incidence of heart failure [1, 2]. Therefore, an in-depth understanding of the pathogenesis of AMI may aid in the discovery of novel immunotherapeutic approaches.

During an AMI attack, cardiac metabolism changes rapidly and hypoxia inhibits the aerobic oxidation of fatty acids, carbohydrates, and amino acids, whereas anaerobic glycolysis is rapidly activated to conserve limited oxygen [3]. Glycolysis is one of the major modes of energy production, where the activation of glycolysis results in rapid ATP production to prevent cardiac parenchymal cell death [4, 5], Furthermore, glycolysis is usually the preferred pathway in immune cells; while it may not be the most efficient method for energy production, it causes rapid activation of immune cells such as neutrophils and monocytes [6]. Neutrophils are the first to be recruited from the blood after AMI, and the inflammatory functions of neutrophils and the formation of neutrophil extracellular traps are mainly dependent on glycolysis [7, 8]. After neutrophil influx, monocytes begin to infiltrate the ischemic heart [9]. In actuality, the monocytes of patients with AMI are hyperinflammatory in instances of increased glycolysis, which leads to nuclear translocation of the glycolytic enzyme pyruvate kinase M2 (PKM2) to facilitate transcription of inflammatory cytokines interleukin (IL)-6 and IL-1β [10], and the inhibition of monocyte glycolysis can attenuate the activation of inflammation [11]. Glycolysis not only provides energy to meet cardiac metabolic needs during AMI, but also has a close relationship with inflammatory cell infiltration, which leads to adverse cardiac remodeling [9]. Therefore, it is important to explore the glycolysis-related markers in immune cells during AMI for immunometabolic treatment.

In this study, we screened glycolysis-related differential genes (GRDEGs) in AMI using bioinformatics, further evaluated the link between key GRDEGs and immune infiltration in patients with AMI, and verified the expression levels of five hub GRDEGs using reverse transcription-quantitative polymerase chain reaction (RT-qPCR) and western blotting. The results of this study may provide novel approaches for the treatment of AMI.

## Materials and methods

### Microarray data

The microarray expression dataset (GSE59867) was obtained from the Gene Expression Omnibus database (https://www.ncbi.nlm.nih.gov/geo/). The dataset includes RNA sequencing results of peripheral blood mononuclear cells (PBMCs) from nine patients 1 day after AMI and another nine patients 4–6 days after AMI. Moreover, 46 stable coronary artery disease (SCAD) controls from GSE59867. Data were sourced from the platform GPL6244 (Affymetrix Human Gene 1.0 ST Array).

### Identification of GRDEGs

R software (version 4.2.2) was used to compare the DEGs between patients with AMI and SCAD controls. The genes with a |log_2_ FC| ≥ 0.1 and an adjusted *p*-value < 0.05 were identified as DEGs. The glycolysis-related gene sets were downloaded from the Molecular Signatures Database (MSigDB) (http://software.broadinstitute.org/gsea/msigdb); a total of 337 glycolysis-related genes (GRGs) were acquired, and by considering their intersection with the DEGs of patients with AMI, we finally identified the AMI-related GRGs.

### Enrichment analysis of GRDEGs

GRDEGs were analyzed by Gene Ontology (GO) and Kyoto Encyclopedia of Genes and Genomes (KEGG) enrichment analyses using the Database for Annotation, Visualization, and Integrated Discovery (DAVID) v.6.8. A *p* value < 0.05 was considered to be statistically significant.

### Identification of hub GRDEGs

The protein–protein interaction **(**PPI) networks of GRDEGs 1 day and 4–6 days after AMI were constructed using PPI network analysis based on the STRING database (https://cn.string-db.org/)and were visualized using Cytoscape software.

### Immune cell infiltration analysis of GRDEGs

CIBERSORT (https://cibersortx.stanford.edu/) was used to compare the proportions of 22 immune cell types between patients with AMI and SCAD controls. To further explore which immune cell type was significantly correlated with key GRDEGs, we performed a Pearson correlation analysis, and the results were visualized using R software.

### Construction and validation of a nomogram for the hub GRDEGs

The “RMS” package (version 6.7-0) was used to construct a nomogram plot based on the hub GRDEGs, which was applied for assessing the clinical incidence of AMI. Calibration curves were plotted to assess the predictive accuracy of the nomograms. Additionally, a decision curve was plotted to evaluate the clinical value of the nomogram. Finally, receiver operating characteristic (ROC) curves were plotted for each gene to assess the probability of AMI occurrence.

### Inclusion of study population and isolation of PBMCs

Four patients diagnosed with AMI and four healthy controls at the Affiliated Hospital of Yangzhou University were enrolled in our study. The inclusion criteria for AMI (type 1, 2, and 3 myocardial infarction) were based on the fourth edition of the universal definition of myocardial infarction [12]: acute myocardial injury accompanied by clinical evidence of acute myocardial ischemia and a diagnosis of acute myocardial infarction was made when an increase and/or decrease in cardiac troponin (cTn) values was detected with at least one value greater than normal, and when at least one of the following was present: symptoms of myocardial ischemia; new onset of ischemic electrocardiographic changes; progression of pathologic Q waves; imaging evidence of loss of viable myocardium consistent with ischemia or localized ventricular wall motion abnormalities; and detection of coronary thrombus by coronary angiography or autopsy (not applicable to type 2 or type 3 myocardial infarction). Patients exhibiting severe hepatic or renal dysfunction, recent severe trauma, autoimmune diseases, glucocorticoid usage, or malignant tumors were excluded. As this study involved human participants, patient informed consent was obtained and the study protocol was approved by the Ethics Committee of the Yangzhou University (YXYLL-2023-133).

Peripheral blood was collected from patients with AMI on the first day after admission. PBMCs were separated using the human PBMC separation solution, and the total cellular RNA and protein of PBMCs were extracted to verify the expression levels of key GRDEGs in patients with AMI.

### RT-qPCR

Total RNA was extracted from the PBMCs of patients with AMI after admission within 24 h using TRIzol Universal Reagent (Tiangen). RNA was reverse-transcribed into cDNA using HiScript III RT SuperMix for qPCR (+ gDNA wiper) (NOVIZAN). Primer sequences are listed in Table 1. The mRNA levels of the hub genes were normalized using *ACTB* as the internal reference gene.

**Table 1:** Primer pairs for RT-qPCR

Genes symbol	Forward (5’-3’)	Reverse (5’-3’)	
*HK2*	CTCCAAATCAGCCTCGGGAC		GCTCCAAGCCCTTTCTCCAT
*PFKL*	GGACCTGGAGAAGCTGCG		GGTCACAGCCATCTCAGCAG
*PKM*	CGAGCCTCAAGTCACTCCAC		GACGAGCTGTCTGGGGATTC
*G6PD*	ACGACGAAGCGCAGACAG		CCGACTGATGGAAGGCATCG
*ALDOA*	AGGGGCTTCAGGTTTCCCTA		TAGTAGCAAGTTCCTGCGGC
*ACTB*-	TGGCACCCAGCACAATGAA		CTAAGTCATAGTCCGCCTAGAAGCA

RT-qPCR, Reverse Transcription-quantitative Polymerase Chain Reaction

### Western blotting

Protein was extracted from human PBMCs using RIPA lysis buffer. Equal amounts of each protein sample were then separated via sodium dodecyl-sulfate polyacrylamide gel electrophoresis, followed by transfer onto polyvinylidene difluoride membranes. After blocking with 5% skimmed milk (Beyotime, Shanghai, China) for 2 h, membranes were incubated with anti-HK2 (1:2000, Cat No. 22029-1-AP, Proteintech, Wuhan, China), anti-PFKL (1:1000, Cat No. 68385-1-Ig, Proteintech), anti-PKM (1:2000, Cat No.15822-1-AP; Proteintech), anti-G6PD (1:1000, Cat No. sc-373,887; Santa cruz, CA, USA), anti-ALDOA (1:1000; Cat No. sc-390,733; Santa cruz), and anti-α-tubulin (1:1000, Cat No. GB11200-100; Servicebio, Wuhan, China) primary antibodies, which were diluted using western blotting specialized primary and secondary antibody diluents (ABSIN, Shanghai, China); overnight at 4 °C. On the following day, the membranes were incubated with the enzyme-labeled secondary antibodies for 2 h. Protein bands were quantified using ImageJ software.

### Statistical analysis

Experimental data are presented as mean ± standard error of the mean (SEM), and statistical analysis was performed using GraphPad Prism (version 8.0) software. For comparisons between the two groups, an unpaired Student’s *t*-test was performed. *p* < 0.05 was considered statistically significant.

## Results

### GRDEGs in AMI

**Fig. 1:**
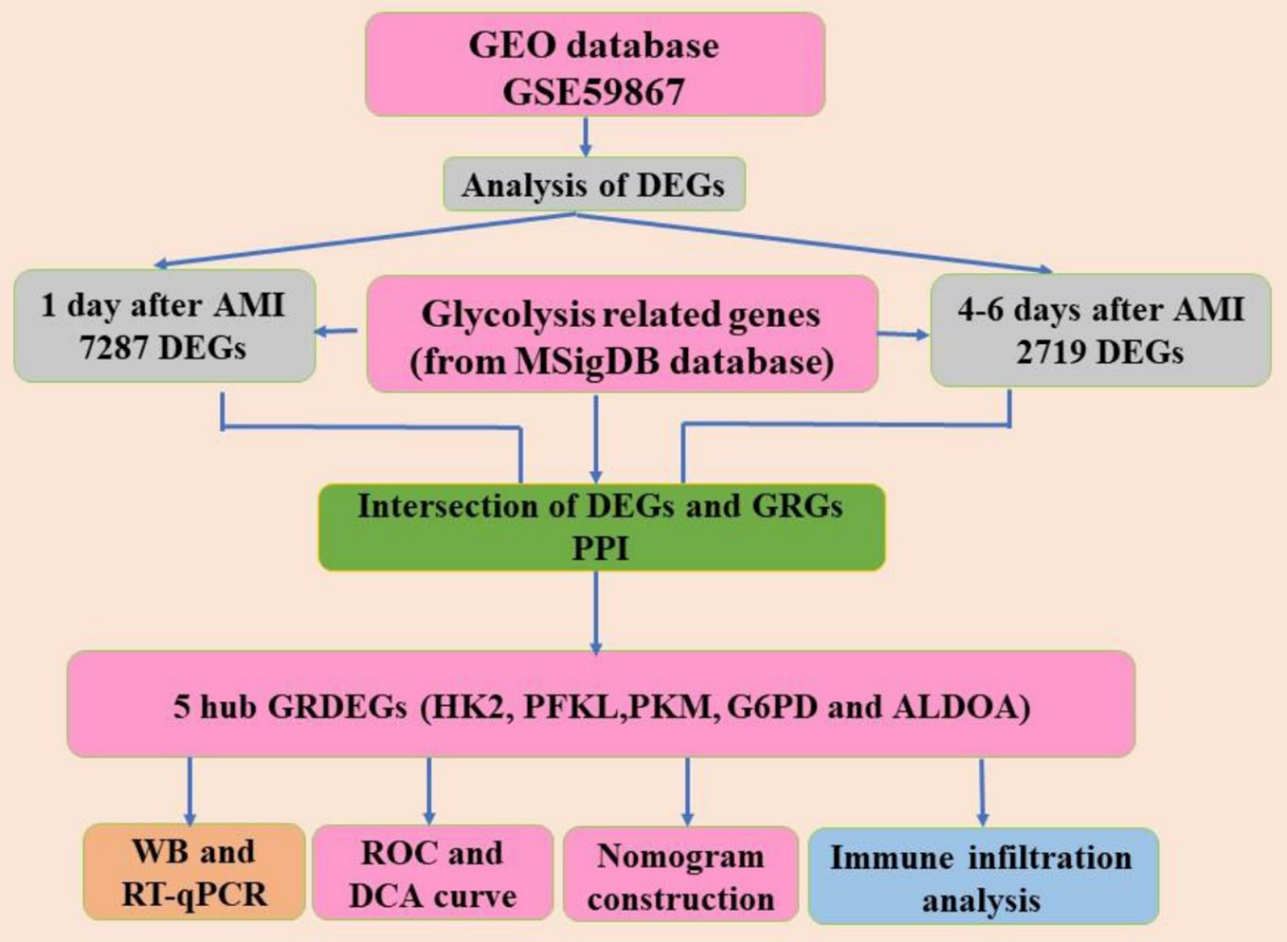
The Flowchart of the study

**Fig. 2:**
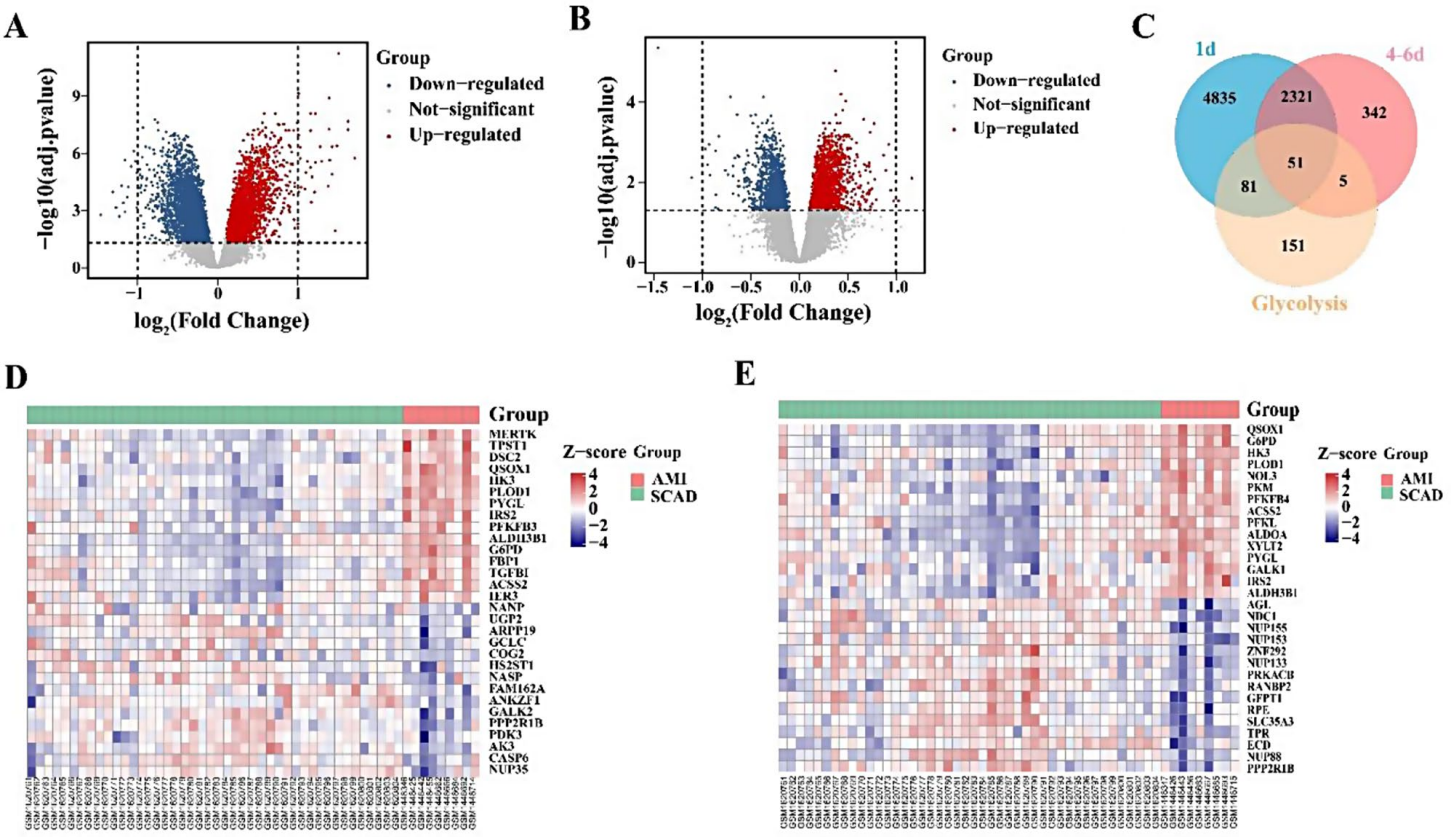
Glycolysis-related differentially expressed genes (GRDEGs) in patients with acute myocardial infarction (AMI). (**A**) Volcano plot of DEGs 1 day after AMI. (**B**) Volcano plot of DEGs at 4–6 days after AMI. The horizontal axis represents the difference in the fold change of gene expression in the AMI group and stable coronary artery disease (SCAD) controls, and the longitudinal coordinate represents the adj. *p*-value of the expression difference. The gray dots represent genes with insignificant differences, red dots represent significantly upregulated DEGs, and blue dots represent significantly downregulated DEGs. (**C**) The common genes between glycolysis-related genes (GRGs) and DEGs 1 day and 4–6 days after AMI. (**D**) Heatmap of differentially expressed GRGs 1 day after AMI based on clustering analysis. (**E**) Heatmap of differentially expressed GRGs 4–6 days after AMI based on clustering analysis

Firstly, we mapped the flow of this study, as shown in Fig. 1. A variety of genes were altered in patients with AMI when compared to SCAD controls. In total, 7,287 and 2,719 DEGs were identified at 1 day and 4–6 days after AMI, respectively, as shown in Fig. 2A, B. We identified 337 GRGs in the MSigDB database; however, after intersecting with DEGs, 132 and 56 GRDEGs were identified at 1 day and 4–6 days after AMI, respectively, as shown in Fig. 2C. Clustering analysis of significantly different GRDEGs 1 day and 4–6 days after AMI indicated that samples from the same group were closely related, as shown in Fig. 2D, E.

### Functional enrichment analysis of GRDEGs

**Fig. 3:**
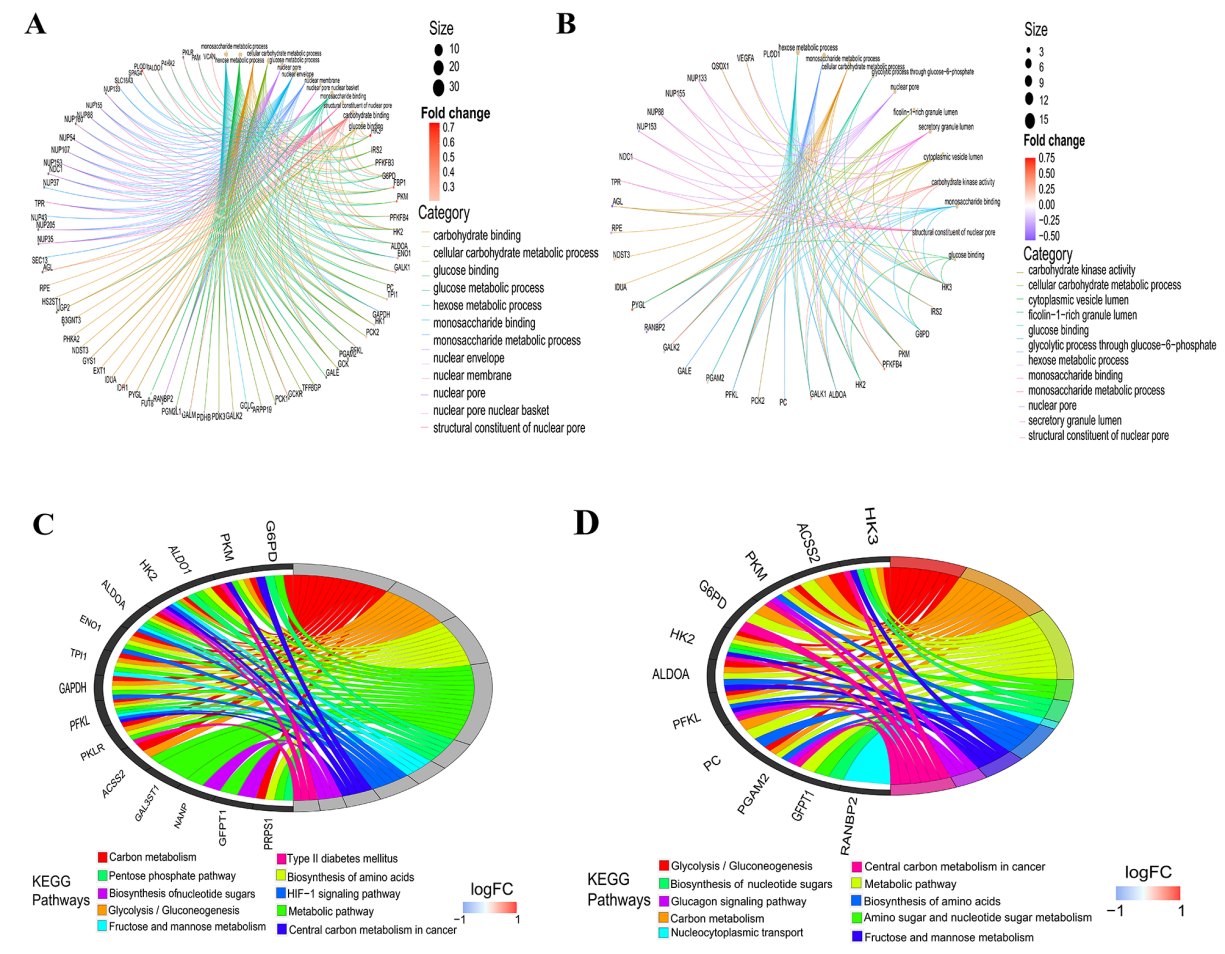
Gene Ontology (GO) and Kyoto Encyclopedia of Genes and Genomes (KEGG) enrichment analysis of GRDEGs. (**A**) GO enrichment analysis of GRDEGs represented in a cnetplot 1 day after AMI. (**B**) GO enrichment analysis of GRDEGs represented in a cnetplot 4–6 days after AMI. (**C**) The relationship between the top 10 enriched KEGG pathways and targets is expressed in a chord plot 1 day after AMI. (**D**) Relationship between the top 10 enriched KEGG pathways and targets is expressed in a chord plot 4–6 days after AMI

GO and KEGG analyses were performed using the DAVID database to further elucidate the biological functions and enrichment pathways of GRDEGs 1 day and 4–6 days after AMI. GO analysis of GRDEGs sorted according to the adj. *p*-value in Fig. 3A revealed four Biological Process (BP) terms, four Cellular Component (CC) terms, and four Molecular Function (MF) terms, such as monosaccharide metabolic processes for BP, nuclear pore for CC, and monosaccharide binding for MF 1 day after AMI. In addition, KEGG analysis indicated that GRDEGs were mainly enriched in glycolysis/gluconeogenesis, carbon metabolism, and metabolic pathways (Fig. 3C). GO analysis at 4–6 days after AMI demonstrated that the GRDEGs were most remarkably enriched in hexose metabolic processes for BP, nuclear pore for CC, and carbohydrate kinase activity for MF, as shown in Fig. 3B. Furthermore, the KEGG results revealed that the GRDEGs were mainly enriched in carbon metabolism, glycolysis/gluconeogenesis, amino acid biosynthesis, and other metabolic pathways at 4–6 days after AMI (Fig. 3D).

### Identification of hub GRDEGs

**Fig. 4:**
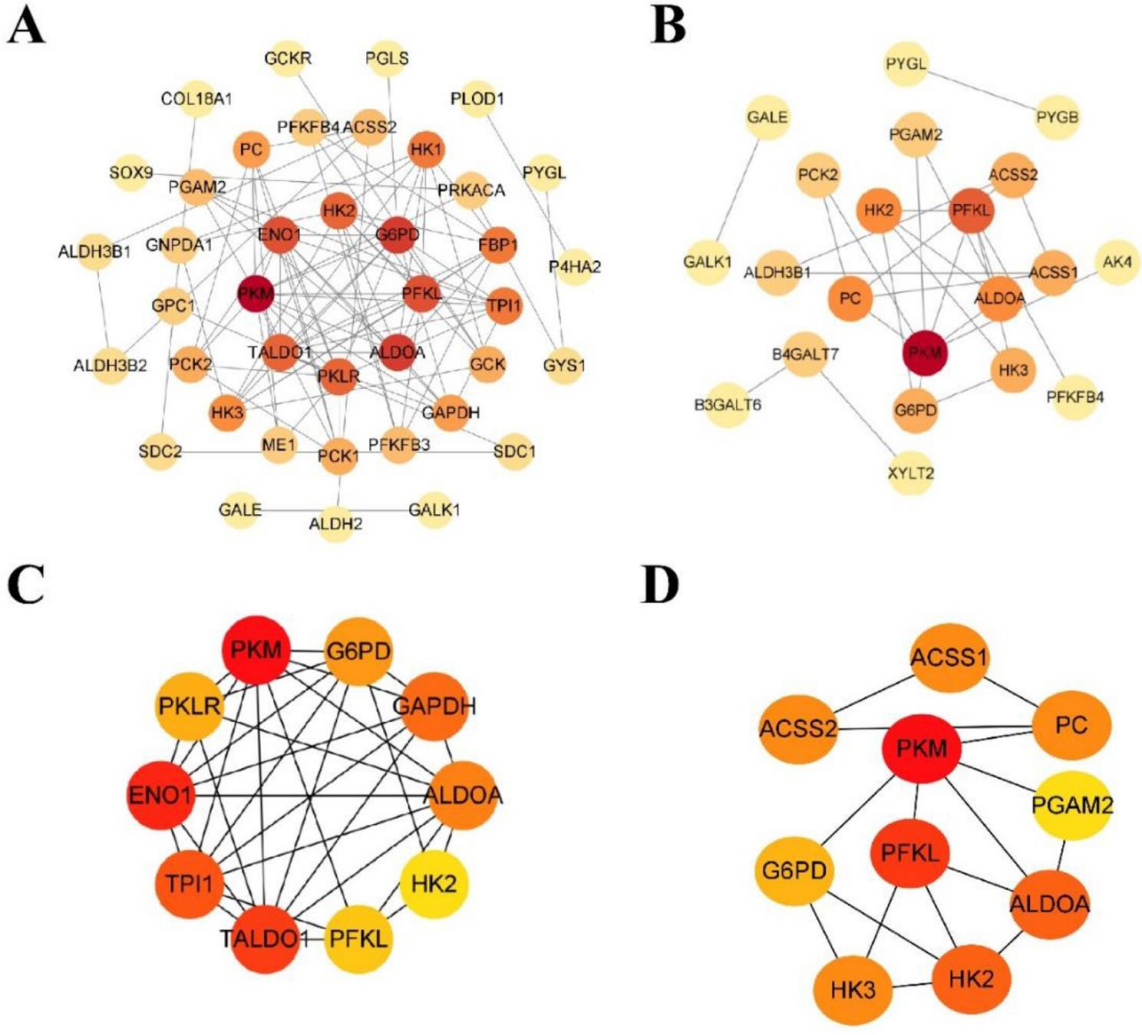
Construction of protein–protein interaction (PPI) network and hub GRDEG analysis. (**A**) PPI network of GRDEGs 1 day after AMI. (**B**) PPI network of GRDEGs 4–6 days after AMI. (**C**) The top five crucial GRDEGs 1 day after AMI were screened based on the degree of nodes. (**D**) The top five crucial GRDEGs 4–6 days after AMI were also screened as above

GRDEGs at 1 and 4–6 days after AMI were analyzed using the STRING database, and a PPI network was constructed (Fig. 4A, B). To further identify hub GRDRGs, the CytoHubba plugin and Maximal Clique Centrality method was used. The top 10 hub GRDEGs 1 day after AMI included *PKM, ENO1, TALDO1, TPI1, GAPDH, G6PD, PKLR, ALDOA, PFKL*, and *HK2* (Fig. 4C). However, at 4–6 days after AMI, the top 10 hub GRDEGs included *PKM, PFKL, ALDOA, HK2, HK3, ACSS1, ACSS2, PC, G6PD*, and *PGAM2* (Fig. 4D). Furthermore, *HK2, PFKL, PKM, G6PD*, and *ALDOA* were common GRDEGs that were upregulated in the AMI group when compared to the SCAD controls at 1 and 4–6 days after AMI.

### Immune infiltration analysis of hub GRDEGs

**Fig. 5:**
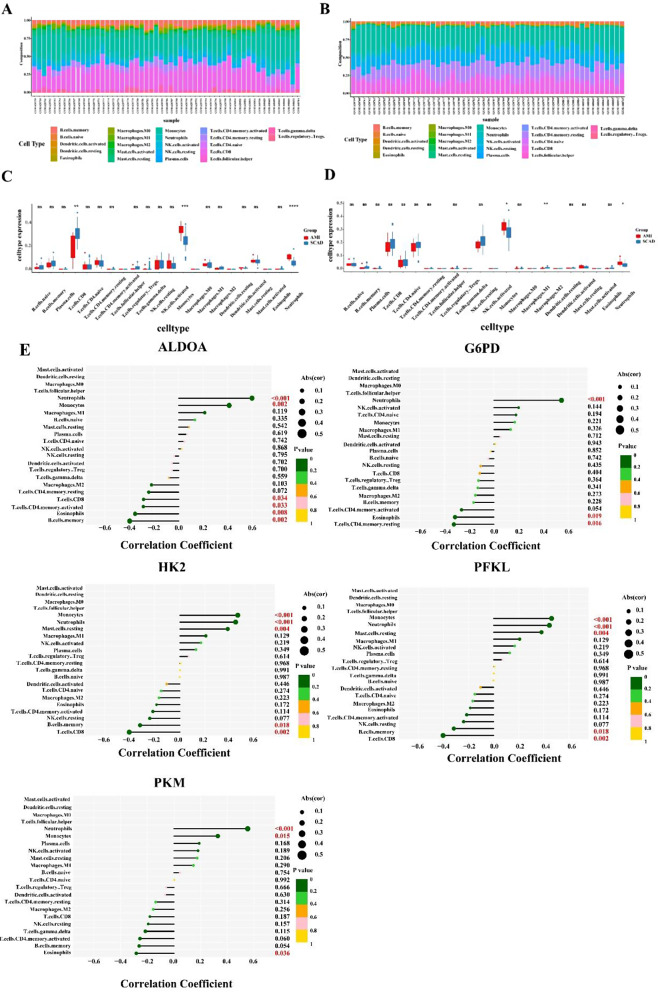
Immune infiltration analysis in patients with acute myocardial infarction (AMI) and SCAD controls. (**A**, **B**) Proportion of 22 immune cell types in patients with AMI and SCAD controls at 1 day (**A**) and 4–6 days (**B**) after AMI. horizontal axis: different samples; longitudinal coordinate: percentage of every immune cell type. (**C**, **D**) Differential immune cell types between patients with AMI and SCAD controls at 1 day (**C**) and 4–6 days (**D**) after AMI. Red represents patients with AMI; blue represents SCAD controls(**E**) Correlation analysis between hub GRDEGs (respectively as *ALDOA, G6PD, HK2, PFKL*, and *PKM*) and infiltrated immune cell types

To further understand the differences between the AMI group and SCAD controls from the perspective of immune infiltration, CIBERSORT was applied to compare the proportion of 22 different immune cell types (Fig. 5A, B). The proportion of monocytes and neutrophils were significantly higher in the AMI group than in the SCAD controls at 1 day and 4–6 days after AMI (Fig. 5C, D). To further explore the link between hub GRDEGs and immune celltypes, we performed Pearson correlation analysis. The results suggested that *HK2, PFKL, PKM*, and *ALDOA* were significantly positively correlated with monocytes and neutrophils, whereas *G6PD* was significantly positively correlated with neutrophils (Fig. 5E).

### Nomogram and ROC curves of hub GRDEGs

**Fig. 6:**
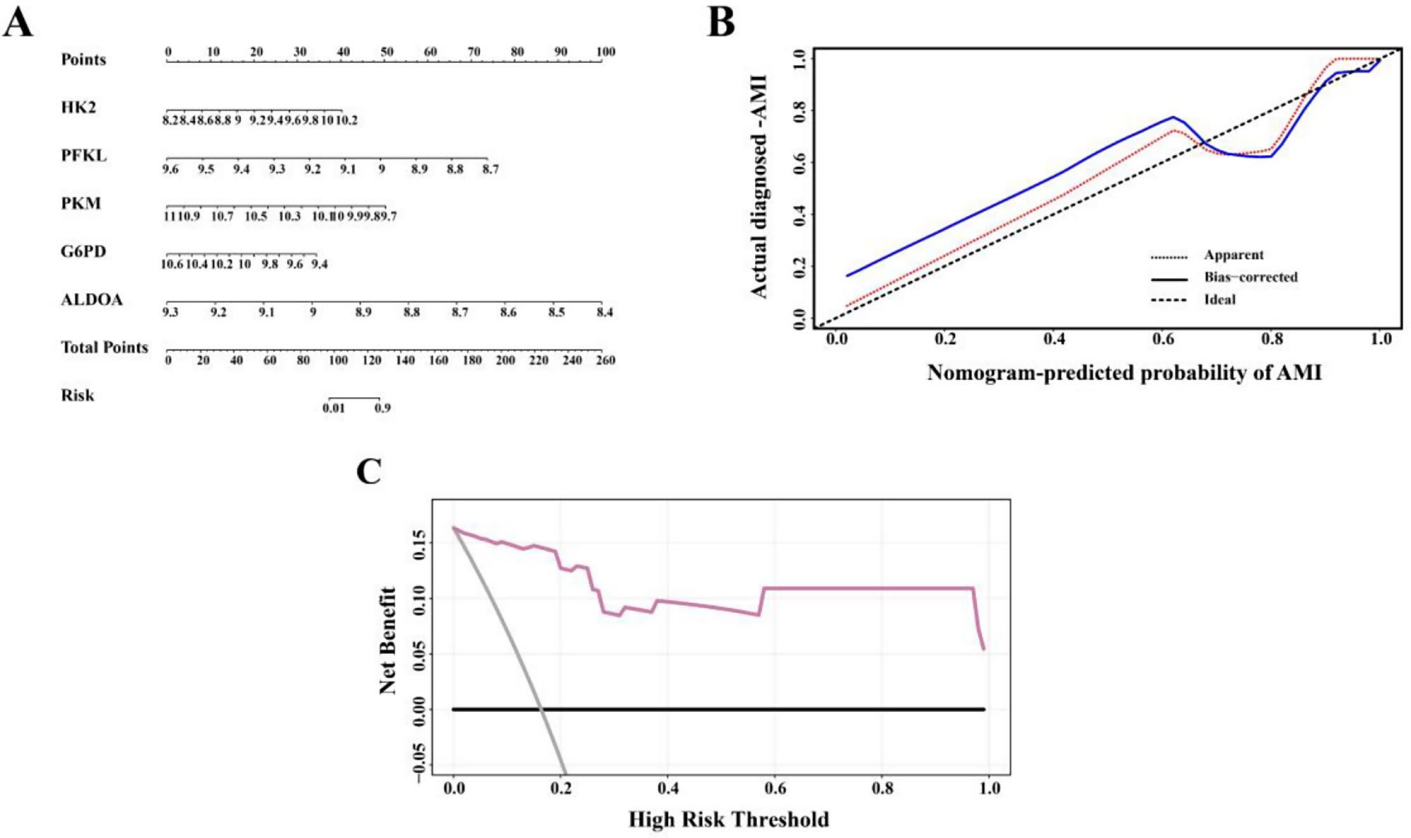
Construction and validation of a nomogram model for AMI diagnosis. (**A**) Nomogram of hub GRDEGs to predict the development of AMI. (**B**) Calibration curve to evaluate the predictive accuracy of the nomogram model. (**C**) Decision curve analysis (DCA) to assess the clinical application value of nomogram model

**Fig. 7:**
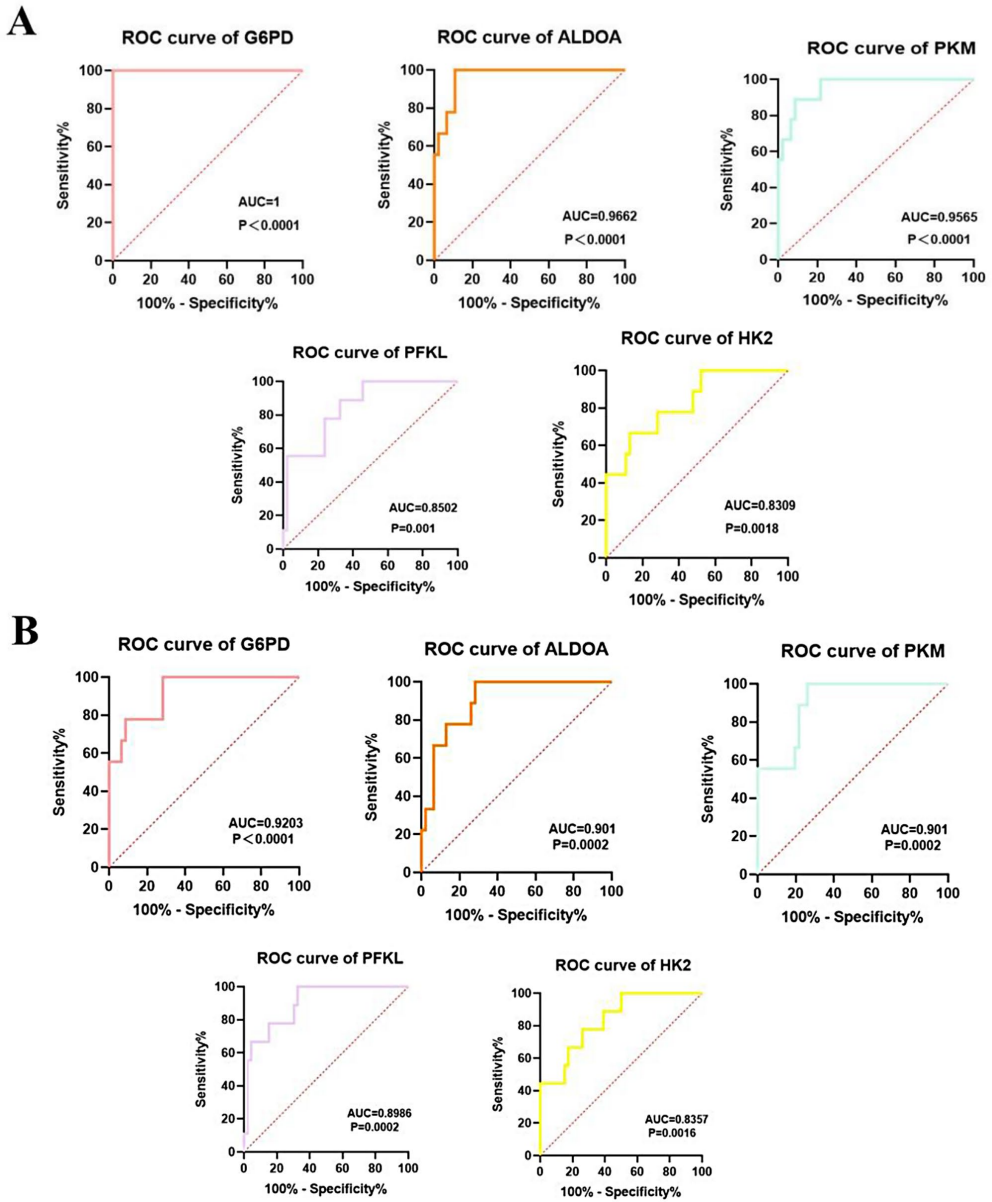
ROC curve for diagnostic efficacy verification of five hub GRDEGs 1 day and 4–6 days after AMI. (**A**) ROC curve for diagnostic efficacy verification of five hub GRDEGs at 1 day after AMI. (**B**) ROC curve for diagnostic efficacy verification of five hub GRDEGs at 4–6 days after AMI. *ROC*, receiver operating characteristic curve

The “RMS” package was used to construct a nomogram based on the hub GRDEGs (Fig. 6A). The calibration curve revealed that the predictive accuracy of the nomogram was high, as the actual AMI risk was close to the predicted risk (Fig. 6B). The decision curve analysis showed that the nomogram curve was higher than the gray line, indicating the high accuracy of the nomogram model (Fig. 6C). These results reveal that the five hub GRDEGs may play important roles in the development of AMI. ROC curves of each hub GRDEG at 1 day and 4–6 days after AMI were constructed, and the area under the ROC curves of *HK2, PFKL, PKM, G6PD*, and *ALDOA* all approached 1 in the AMI group when compared with that in the SCAD controls (Fig. 7A, B). The results indicate that the five hub GRDEGs are able to distinguish patients with AMI from SCAD controls.

### Validation of hub GRDEGs

**Fig. 8:**
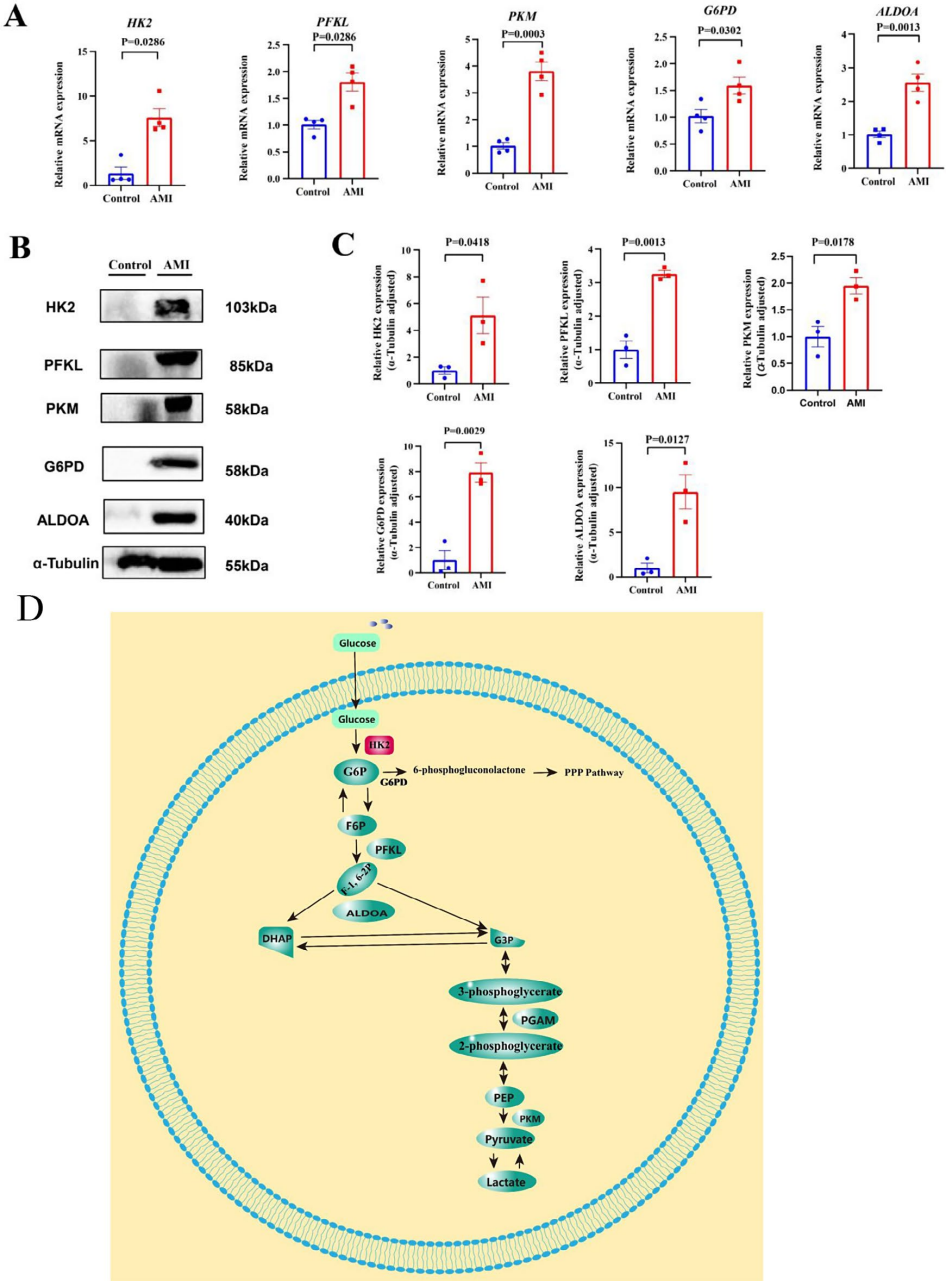
Validation of hub GRDEGs expression at the mRNA and protein levels. (**A**) The mRNA levels of *HK2*, *PFKL*, *PKM*, *G6PD*, and *ALDOA* at 1 day after AMI in patients with AMI and SCAD controls. Expression levels were standardized to *ACTB* expression. (**B**, **C**) Representative western blot and quantitative analysis of HK2, PFKL, PKM, G6PD, and ALDOA at 1 day after AMI in patients with AMI and SCAD controls. Expression levels were standardized to that of α-tubulin expression. A two-tailed unpaired Student’s t-test was applied to compare two groups. Results are expressed as means ± standard error of mean (SEM) for reverse transcription-quantitative polymerase chain reaction (RT-qPCR; *n* = 4) and western blotting (*n* = 3), *p < 0.05; **p < 0.01 and ***p < 0.001 vs. SCAD controls. (**D**) The pathophysiological schemes of hub GRDEGs interaction

To validate the bioinformatics analysis results of the GRDEGs, we assessed the PBMCs isolated from patients with AMI and SCAD controls. The RT-qPCR results revealed that the mRNA expression levels of *HK2, PFKL, PKM, G6PD*, and *ALDOA* in the AMI group were significantly higher than those in SCAD controls (Fig. 8A). We further verified the five hub GRDEGs at the protein level using western blotting (Fig. 8B, C). The results revealed that the protein expression levels of HK2, PFKL, PKM, G6PD, and ALDOA in the AMI group were higher than those of the SCAD controls (*p* < 0.05). Therefore, the RT-qPCR and western blotting results supported the bioinformatics analysis results. The interactions between five hub GREDGs are also shown in Fig. 8D, with the five hub GREDGs working in concert with each other to play a role in AMI occurrence.

## Discussion

Under normoxia, the heart produces approximately 90% of its ATP through mitochondrial oxidative catabolism, with glycolysis producing only 5–10% of the ATP. Glycolysis only becomes the major source of ATP during cardiac ischemia [13]. When the heart is exposed to ischemia and hypoxia, a cardiomyocyte death program is initiated [14]. Necrotic cardiomyocytes release danger-associated molecular patterns, which interact with homologous pattern recognition receptors expressed on circulating leukocytes to activate immune responses [15]. Innate immune cells, including monocytes and neutrophils, will experience varying oxygen and metabolite availability as they are trafficked from the peripheral blood to the damaged heart [16, 17]. Glycolysis is the pathway that is typically preferred by immune cells; although it is not the most efficient pathway for generating energy, it provides ATP support for rapid immune cell activation [9], thus glycolysis and immune cell metabolism are linked, and further research into this link is warranted.

Although there have been many studies on the correlation between AMI and glycolysis, to the best of our knowledge, there have been no studies using bioinformatics analysis to link AMI with glycolysis. In this study, we isolated the PBMCs of patients with AMI, analyzed the DEGs associated with AMI, and further intersected them with glycolytic genes to ultimately obtain GRDEGs in AMI, which were associated with immune cell infiltration. Thus, we analyzed the role of GRDEGs in the activation of immune cells during the development of AMI, which might provide targets for the immunometabolic treatment of AMI.

After downloading the GSE59867 dataset, 7,287 and 2,719 DEGs were identified 1 day and 4–6 days after AMI, respectively. We obtained a list of GRDEGs after intersecting with GRGs, and KEGG enrichment analysis revealed that these genes were mainly enriched in glycolysis/gluconeogenesis and metabolic pathways. The PPI network and CytoHubba plugin were used to screen the hub genes at 1 day and 4–6 days after AMI. Finally, we obtained a list of five genes, including *HK2, PFKL, PKM, G6PD*, and *ALDOA*, that were considered as intersecting GRDEGs at 1 day and 4–6 days after AMI.

*HK2* encodes the first key enzyme in the glycolytic pathway; enzyme that phosphorylates glucose to glucose-6-phosphate (G6P) [18]. Studies have shown that increased *HK2* activity after AMI is correlated with oxidative stress and increased left ventricular end-diastolic volume suggesting that *HK2* may be involved in post-AMI heart failure [19]. Phosphofructokinase 1 (*PFK-1*) is responsible for the conversion of fructose-6-phosphate to fructose-1,6-bisphosphate. *PFKL* is the liver isoform of *PFK-1* [20]. *PFK-1* expression is mainly regulated by fructose-2,6-bisphosphate (F-2,6-BP). Studies have revealed that a long-term stable increase in F-2,6-BP can increase glycolysis, which may be responsible for promoting myocardial hypertrophy and fibrosis in failing hearts [21]. *G6PD* encodes a key enzyme in the oxidized pentose phosphate pathway. *G6PD* activity has been shown to maintain cellular glutathione levels and prevent oxidative stress-induced cardiac insufficiency [22]. *PKM2*, which encodes an isoform of *PKM*, is a key mediator of the Warburg effect, and its low activity state is critical for maintaining aerobic glycolysis [23, 24]. *PKM2* upregulation has a critical effecton ATP synthesis in the infarcted myocardium [25]. *ALDOA* encodes an important glycolytic enzyme that plays pivotal roles in glycolysis, gluconeogenesis, and energy balance [26]. Studies have shown that high expression of *ALDOA* correlates with cardiac inflammation and fibrosis [27, 28]. The above results suggest that the five key GRDEGs are closely associated with cardiovascular diseases.

The five hub GRDEGs were then subjected to immune infiltration analysis. The results showed that *HK2, PFKL, PKM*, and *ALDOA* were significantly positively correlated with monocytes and neutrophils, whereas *G6PD* was significantly positively correlated with neutrophils, suggesting that glycolysis is closely related to the inflammatory infiltration of immune cells after AMI, which was consistent with prior studies [9]. We also analyzed the monocyte and neutrophil ratios in the blood test results of study participants, and confirmed that the monocyte and neutrophil ratios of patients with AMI were significantly higher than those of the control group, and study of single-cell RNA sequencing of PBMCs from AMI also demonstrated that the circulating immune cells of patients with plaque erosion is mainly associated with monocyte amplification and neutrophil activation [29]. In summary, we can conclude that monocytes and neutrophils do play an important role in the mechanism of AMI pathogenesis. We then applied ROC curve analysis to assess the value of each marker in predicting AMI occurrence, and the results suggested that all five genes had a high predictive value for 1d-AMI and 4-6-d AMI occurrence. The nomogram model was constructed based on the five hub GRDEGs between patients with AMI and the control group, including *HK2, PFKL*, *PKM*, *G6PD* and *ALDOA*. The results indicated that the five hub GRDEGs were able to distinguish patients with AMI from SCAD controls. Therefore, each hub GRDEGs and the nomogram model for all five hub GRDEGs were able to distinguish between patients with AMI and control participants, which demonstrated favorable potential for clinical application.

Collectively, our study identified potential immunotherapeutic targets for the treatment of AMI, and implemented RT-qPCR and western blotting methods to verify the key genes. In spite of this, this study had some limitations. First, the sample size of patients with AMI was small and should be increased in the future. In addition, we did not fully identify the connection between hub GRDEGs and immune cells. Moreover, as the clinical collection of peripheral blood from study subjects could not always be completed according to our timetable, we only collected routine peripheral blood within 24 h of admission and did not collect PBMCs at 4–6 days after AMI for validation. Finally, we haven’t explored the interconnections between 5 hub GRDEGs, which can be further refined in future studies.

## Conclusions

This study aimed to identify and experimentally validate hub GRDEGs in AMI. Our bioinformatics results suggested that five hub genes (*HK2, PFKL, PKM, G6PD*, and *ALDOA*) are closely associated with glycolysis and immune infiltration in AMI and may be potential immunotherapy targets for AMI. The connection between hub GRDEGs and immune cells requires further exploration in future studies.

### Electronic supplementary material

Below is the link to the electronic supplementary material.

Supplementary Material 1

## Data Availability

Public datasets were analyzed in this study. These data can be found: The Gene Expression Omnibus database (GEO): https://www.ncbi.nlm.nih.gov/geo/. The Molecular Signatures Database (MSigDB): http://software.broadinstitute.org/gsea/msigdb. The STRING database: https://cn.string-db.org/. CIBERSORT: https://cibersortx.stanford.edu/.
