# Supplementary material for: Identification of hub glycolysis-related genes in acute myocardial infarction and their correlation with immune infiltration using bioinformatics analysis
Source: BMC Cardiovasc Disord. 2024 Jul 10;24:349. doi: 10.1186/s12872-024-03989-7 (PMC11234719; doi:10.1186/s12872-024-03989-7)
Supplement: Supplementary file 1 — Supplementary Material 1 [file 12872_2024_3989_MOESM1_ESM.docx]

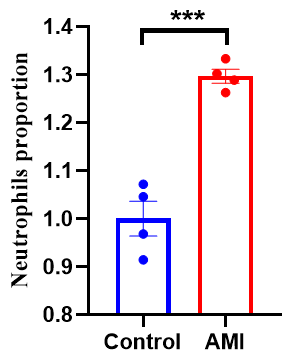

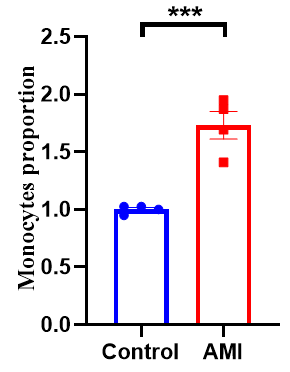


**Figure S1** The monocyte and neutrophil ratios in the blood tests of patients with AMI and controls.

**Table S1** The baseline characteristics of patients with AMI and controls.

| **Characteristic** | **Control (n=4)** | **AMI (n=4)** | ***P* Value** |
| --- | --- | --- | --- |
| SBP, mmHg | 126.75±10.69 | 138.50±31.81 | 0.51 |
| DBP, mmHg | 76.25±13.72 | 82.25±15.22 | 0.58 |
| LVEF(%) | 66.25±6.29 | 65.50±3.70 | 0.84 |
| LVESD(mm) | 32.00±2.16 | 34.50±5.20 | 0.41 |
| Total protein, g/L | 74.27±5.56 | 65.65±6.75 | 0.096 |
| Serum albumin, g/L | 45.58±3.29 | 39.83±4.86 | 0.098 |
| Serum globulin, g/L | 28.70±5.07 | 25.83±2.68 | 0.36 |
| WBC, 10^9^ /L | 7.91±2.41 | 8.82±2.80 | 0.64 |
| RBC, 10^12^/L | 4.43±0.41 | 4.74±0.74 | 0.49 |
| LDL-C, mmol/L | 2.68±1.22 | 2.90±0.12 | 0.76 |
| Lp(a), mg/L | 116.33±141.15 | 355.62±230.38 | 0.15 |
| ALT(U/L) | 22.88±6.46 | 29.00±8.29 | 0.29 |
| AST(U/L) | 21.43±4.78 | 34.25±6.85 | 0.02 |
| Scr, umol/L | 55.63±12.37 | 69.30±18.22 | 0.26 |
| UA, umol/L | 289.38±53.86 | 315.20±150.38 | 0.76 |

ALT, alanine aminotransferase; AST, aspartate aminotransferase; DBP, diastolic blood pressure; LVEF, left Ventricular Ejection Fractions; LVESD, left ventricular end-systolic volume; LDL-C, low-density lipoprotein cholesterol; Lp(a), Lipoprotein (a); RBC, red blood cell; SBP, systolic blood pressure; Scr, serum creatinine; UA, uric acid; WBC, white blood cell.
